# Feasibility and Application of Local Closed-Loop Materials to Produce Compressed and Stabilized Earth Blocks

**DOI:** 10.3390/ma17133358

**Published:** 2024-07-07

**Authors:** Catalina Reyna-Ruiz, José Manuel Gómez-Soberón, María Neftalí Rojas-Valencia

**Affiliations:** 1 Barcelona School of Architecture, Polytechnic University of Catalonia, 649 Diagonal Avenue, 08028 Barcelona, Spain; catalina.reyna@upc.edu; 2 Department of Architecture Technology, Barcelona School of Building Construction, Polytechnic University of Catalonia, Av. Doctor Marañón 44-50, 08028 Barcelona, Spain; 3 Institute of Engineering, Coordination of Environmental Engineering, National Autonomous University of Mexico, Av. Universidad 3000 C.P., Mexico City 04510, Mexico; mrojasv@iingen.unam.mx

**Keywords:** blocks of stabilized earth, compressed, construction and demolition, glass, recycled, second generation materials, waste

## Abstract

The validation of a feasible application for the production of sustainable bricks with local materials in humid and hot climates, which would allow the current housing needs of a constantly growing population with scarce economic resources to be met while also reducing energy inputs for climate control, is a current challenge without a definitive solution. Therefore, this research studied the incorporation of local aggregates and two second-generation materials to produce lime-stabilized Compressed Earth Blocks (CSEBs) using a semi-automatic machine for their manufacture. An initial matrix was designed as a baseline, and three more were developed with variations to incorporate second-generation materials individually and as mixtures. The stabilizer was added in concentrations of 5, 10, and 15%, resulting in a total of 12 batches of CSEBs. Eleven of the studied batches exceed the normative limits for simple compressive strength and initial water absorption coefficient. The best result of simple compressive strength was obtained in two batches of the same matrix that used construction demolition waste (CDW), reaching 4.3 MPa (43% above the minimum limit established by the most restrictive regulations and 115% above the least restrictive). It was possible to produce sustainable bricks in situ with average ambient temperatures of 32 °C and relative humidity of 91%.

## 1. Introduction

In 2022, the world’s population reached eight billion [1], having multiplied 2.6 times [2] in the last six decades. The three most populous countries in the world (in descending order) are: India, China, and the United States (USA) [2]. If we compare these countries for the same year, the energy consumption of the US and China remained above 20,000 TWh, while India only used 10,000 TWh [3]. Residential energy consumption in the U.S. accounts for 16% of its total energy consumption [4], and more than half of that energy goes to HVAC [5].

Despite the fact that CO_2_ emissions related to energy production have decreased to below 2005 levels throughout the U.S., thanks to the expansion of the electric grid, improved equipment efficiency, and increased generation of emission-neutral energy, the total energy-related CO_2_ emissions in 2022 were in the order of 5 billion metric tons (MTm) [6].

A report by the National Weather Service on the history of hurricanes in Louisiana concluded that Hurricane Katrina (August 2005) was the worst natural disaster in U.S. history, with most of the damage reported in New Orleans and the Gulf Coast of the state of Mississippi, with 1577 lives lost in the state of Louisiana [7]. Considering that CO_2_ is one of the main greenhouse gases, the increase in its emissions leads us to expect greater negative effects in general [8].

It is essential to find building materials whose manufacture requires less energy and that have good insulation characteristics and resistance to wind forces and flooding. The Compressed and Stabilized Earth Block (CSEB) is a possible candidate as a building material with these characteristics. Previous research has studied some interesting aspects of CSEBs, comparing them with different wall systems [9] and establishing that these materials have good thermal insulation. In addition, they are highly efficient environmentally, as during their manufacture [10], they emit 75% less CO_2_ than traditional oven-fired blocks [11]. It has been established that it is possible to build homes with CSEBs that can withstand the wind loads of category 5 hurricanes and even EF3 tornadoes [12].

CSEBs are a “modern” material compared to the traditional hand-molded adobes that are part of the historical construction heritage in the Americas. In the case of Mexico, one study used modern microscopy techniques, both optical and electronic, to identify the crystalline and amorphous components of the adobe of the Great Pyramid of Cholula in Puebla (built between the years 30 and 450 CE) [13]. Another study analyzed adobe samples from excavations in Huichapan, Hidalgo (birthplace of the Xajay Culture) using X-ray diffraction and demonstrated their low crystallinity. Through stratigraphic analysis of archaeological materials, it was possible to date the occupation of these cultural developments to between 600–1200 CE [14]. Similarly, earthen construction techniques have been present in Europe since ancient times, as indicated by research from Spain that identified 293 adobe constructions in the Iberian Peninsula [15].

In order to increase the sustainability of CSEB manufacturing, there is a tendency to incorporate natural additives and by-products from other industries or processes, materials that would otherwise be considered a waste. Consequently, a wide range of materials have been used, including: wood cutting waste, excavation process waste, recycled aggregates, natural mucilage [16,17], granulated slag [18], sawdust ash [19], fly ash [20], waste from the paper industry [21], biopolymers, polysaccharides, chitosan, thermo-gelatinous biopolymers, guar and xanthan gums, carrageenan, alginate, lipids, proteins, tannins, lignin and lignin sulfonate [22], coconut fibers [23], alkali-treated date palm fibers [24,25], polypropylene fibers [26], sugarcane bagasse [27], and oil palm residues [28].

Other studies have added residues and by-products to the mortar, for example, replacing 15, 30, 60, and 100% of the usual aggregate with recycled glass aggregate [29]. Other researchers have used aggregates of recycled concrete, glass, recycled ceramics [30] and polyhydroxyalkanoates (produced by bacteria that digest glycerol, a by-product of biodiesel processing) in both mortar and surface coatings of Compressed Earth Blocks [31], as well as by-products of bacteria growing in a lysogeny broth with added iron [32].

This research studied the mechanical fabrication of CSEBs cured in the hot and humid climate of southern Louisiana. A base additive was used as a standard, and three more additives were tested with the addition of second-generation materials and lime as a stabilizer. Stabilization of earth-based materials improves their resistance to the detrimental effects of water [15]. Lime in the presence of water reacts with the clay fraction to produce calcium hydroxide, which allows the clay to stabilize through carbonation reactions aided by the absorption of carbon dioxide present in the air [33]. Characterization tests, which are detailed below, were carried out on both aggregates and manufactured blocks. The methods used and the results obtained provide a viable technical option for the aforementioned climate, and local materials are used with the aim of moving towards a decarbonized and more sustainable construction industry.

## 2. Materials and Methods

### 2.1. Aggregates and Stabilizers

The following materials were sourced from local commercial aggregate suppliers in Louisiana. In all cases, the materials were obtained from a single batch. All aggregates were screened using a metal sieve with 6.35 mm openings.

- Stiff clay soil (SCS);
- Spill way dirt (SWD).

The two above-mentioned soils are excavated native clays, free of wood, debris, and rocks, which are removed by the vendor.

**Figure 1:**
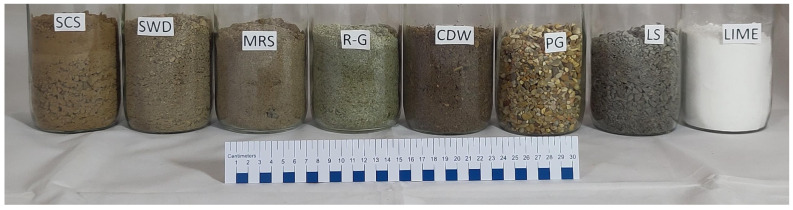
Aggregates and stabilizers.

- Mississippi River sand (MRS).
- Recycled glass (R-G) sand, from a mix of colored bottles and jars.
- Construction demolition waste (CDW), originating from demolished structures, construction waste, and other sources of crushed concrete.
- Pea gravel (PG), small stones of rounded and smooth edges as a result of natural weathering.
- Limestone (LS) # 8 is a sedimentary material with angular edges.
- High calcium hydrated lime (Lime), tradename Lhoist, has a composition of >90% calcium hydroxide (CAS# 1305-62-0), <3% magnesium oxide (CAS# 1309-48-4), and <2% crystalline silica (CAS# 14808-60-7) with an apparent density of 400–700 kg/m^3^. Figure 1 shows the aggregates and the stabilizer used.

### 2.2. Characterization of Aggregates

**Figure 2:**
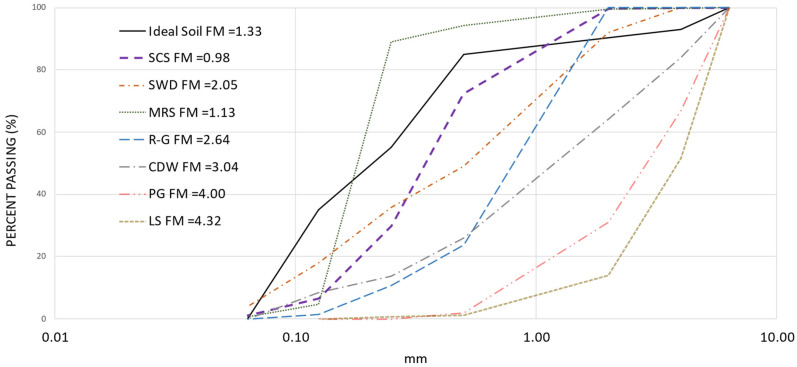
Particle size distribution of aggregates and ideal reference soil and their finesse module.

Particle size distribution (PSD) was determined using woven wire mesh test sieves according to the ISO 2591 analytical protocol [34]. Sieves numbers 5, 10, 35, 60, 120, and 230 were used, corresponding to mesh apertures of 4000, 2000, 500, 250, 125, and 63 μm respectively. The ideal particle size distribution varies depending on the stabilizer for CSEB production. For example, for the use of lime, it is 35% clay, 20% silt, 30% sand, and 15% gravel [35]. In another study that analyzed the combined fraction of clay and silt, it had a value of 57.08%, with 42.12% sand and 0.8% gravel [36]. A third case study cited the international recommendation for construction with earth, which combines gravel and sand in a common fraction of 65%, with 26% clay and 9% silt [37]. In another case, a soil mixture with 15.9% gravel and 47.2% sand was used, where clay and silt combined made up 36.9% [38]. The third and fourth approaches cited above are very similar, while the first and second coincide with the combined fraction of clay and silt. In another study, soil with a silt fraction of 46% and 16% clay was used [39]. Considering the previous studies, in this research the use of 55% combined fraction of clay and silt was established as a good option. To compare the PSD of each aggregate with the “ideal soil”, it was assumed that the percentage of the fractions was the percentage of mass retained in each sieve size. Figure 2 shows the PSD and an approximation to the Fineness Modulus (FM) (descriptor of the material gradation obtained by adding the percentages of the materials in the sample that are retained by certain sizes of standard sieve numbers 4, 8, 16, 30, 50, and 100 and dividing by 100) [40]. For the present study, this allows the specific comparison of the aggregates used, and the formula was applied for the above sieve sizes as indicated above.

In general terms, it can be seen that most of the profiles, thicker soils, are below that of the so-called “ideal soil”. It is also evident in two profiles, MRS and LS, which have a staggered transition (the most critical is MRS, absence of sizes). In terms of approximation to FM, the SWD, R-G, and CDW soils are within the usual ‘fine’ limits—between 2.2 and 3.2 [41]. It will be necessary to consider the above when making aggregate combinations for research to verify that the resulting samples have the workability, uniformity, and compaction capacity that will provide specimens with adequate volumetric, physical, and mechanical properties.

**Figure 3:**
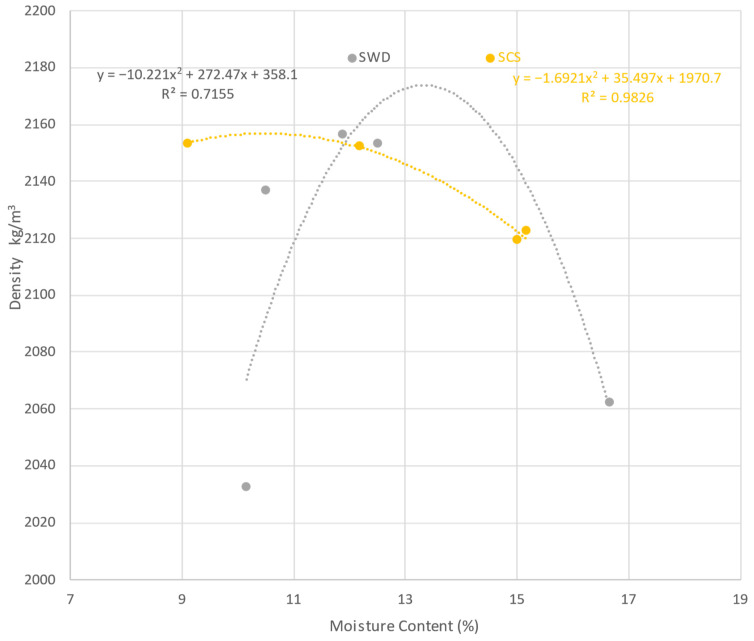
Proctor test for soils.

The Proctor test (determination of soil moisture content producing maximum density) was performed in accordance with NF P 94-093 [42], using a 2.5 kg soil compaction hammer (Gilson model HM-550) and a set of 10 cm diameter soil density molds (Gilson model HMA-110) from Gilson Company Inc. (Lewis Center, OH, USA). This test is reported only for SCS and SWD, as they are the only aggregates identified as soils, establishing density and moisture values of 2157 kg/m^3^ with 12.05% and 2174 kg/cm^3^ with 13.2%, respectively. The average value of these densities is 14% higher than the average densities reported in a previous study using five types of soils [43]. In the aforementioned research, mean densities between 1820 and 1980 kg/cm^3^ were obtained, while their humidity was in a similar range (11.25 and 15.2%) [43]. In another study, a density of 1680 kg/cm^3^ was established with a 28% higher average soil density than 9% of the soil in this study, coinciding with the SCS soil [44]. Finally, in the third study of natural dredged sediments, the density ranged from 1515 to 1665 kg/cm^3^, but in this case the moisture range was 23.5 to 27%; the average is twice as high as the average for SCS and SWD soils [45]. In this study, a compiled table—from six different citations—is presented, showing the recommended values of these properties for Compressed Earth Blocks (CEBs) [45], and establishing the global density range between 1631 to 2345 kg/cm^3^ and from 5 to 19% for humidity. SCS and SWD soils are within these limits. Figure 3 shows the results with polynomial trend lines, as well as the respective equations and values of the correlation coefficient (R^2^).

The maximum compaction density values found by the polynomial equations of SWD and SCS are very close, with a difference of 0.5%, while the difference in humidity is 11%. However, the fact that the correlation coefficient of the polynomial equation of SWD is 0.7155 while that of SCS is 0.9826 could indicate that the mathematical value has a deformation. There could be another type of equation that allows a better fit for SWD, but in this case it would no longer be valid for comparing both soils. 

**Figure 4:**
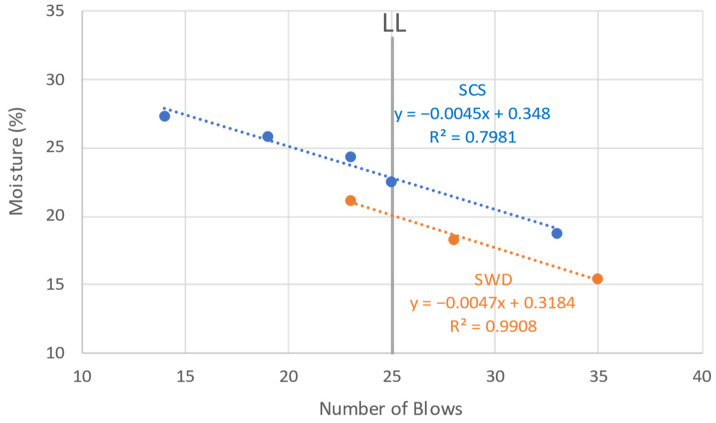
Liquid limit (LL) test results of the studied soils.

Atterberg limits are indicators of plasticity (i.e., liquid limit (LL), plastic limit (PL), and plasticity index (PI)), which is the deformation capacity of a material without breaking [46]. Like the Proctor test, these limits are prescribed for soils and were determined only for the same aggregates according to the DOTD TR 428 method [47], obtaining for SCS: LL = 23.55%, PL = 8.1%, PI = 15.45% and for SWD: LL = 20.09%, PL = 11.77%, PI = 8.3%. The classification of soil types based on the PI proposes that values of 7 to 17% correspond to clayey silt soils of medium plasticity and cohesiveness. Both study soils are within this range [48]. From other previous studies reviewed, natural soil obtained values similar to the SCS: LL = 26.9% and PI = 17.5% [49]. Figure 4 shows the LL values (indicated by the moisture content at 25 blows).

The liquid behavior of each soil, determined by the dispersion of the points, shows a similar trend between the two, since the adjusted lines are close to each other despite the fact that the coefficient of determination of SWD is 20% better than that of SCS. The spacing of the lines is equivalent to the difference between the LLs of each floor, which is 15%.

The shrinkage limit (SL) test was also performed for the two studied soils (AS 1289 C4.1-1977 method [50]), using rectangular stainless-steel molds of 19 × 11 × 254 mm (internal measurements). The SL was considered as the reduction in size in the longest section of the soil sample, dried in an oven (General Electric model JGBS66REKSS) at a temperature of 110 °C, and starting the test with a moisture content equal to LL. Four series were performed per soil type, and the mean values were 8.3% for SCS and 5.1% for SWD. These results are located in the lower range compared to a compilation of 265 test results from 26 previous studies in which SL values ranged from 0.5 to 32% and where clay soils experienced more significant volumetric changes (such as shrinkage and fissures) compared to silty soils [51]. Therefore, lower shrinkage can be associated with the silt content exceeding the clay content in a sample.

### 2.3. Matrix Design

The base mixtures for the elaboration of the blocks were designed in accordance with the recommendation of CRAterre, the UNESCO Chair of “Earthen Architecture, Building Cultures, and Sustainable Development” [36], which is for a granulometric profile previously defined as the PSD of an ideal soil. To achieve this, the silt and clay fractions came from a mixture of two local clay soils with equal proportions—similar FMs—(SCS and SWD), plus sand from the Mississippi River. Two types of rock aggregates were used for the gravel fraction (PG and LS). Lime was added as a stabilizer in three different concentrations: 5, 10, and 15% by total weight of the materials. In other research, this lime content has been limited to below 10% [49,52] or as high as 12% [24,26]. In this research, the maximum limit established by the NMX-C0508-ONNCCEE-2015 standard is exceeded, to 15% [53].

All materials were protected from rainwater. Based on the previous determination of the moisture content, a correction was made, when required, to keep it equal to 10%. As the material was mixed, the calculated correction water was added, and then the actual moisture content was determined, with an average of two tests in each sub-batch mixed. The final compositions were adjusted with the actual humidity analyzed. Once the percentage of correction water and stabilizer were added to the mix, the required amounts of soils and aggregates were added. Table 1 shows the composition of each matrix and its three batches in percentage by weight.

This research aimed to control the variables of the materials and their mixing within the constraints of an in situ production of CSEB in a hot and humid climate. However, despite efforts to keep the water content at about 10% [54], the actual figures changed in order to achieve the ideal workability of the mixture. Similarly, other researchers also used water concentrations of 11 to 13% and lime concentrations of 8 to 12% [25], as well as 13 to 17% water for 5 to 12% lime [36] or, in another case, 12% water in all lime preparations [55].

The first matrix, or base, of this research is described using the following nomenclature: base (B), followed by a number indicating the percentage of lime (B5, B10, B15). For these three batches, the soil/sand ratio (So/Sa) was 0.87, 0.86, and 0.96, respectively. For the following mix designs, river sand (MRS) was replaced with recycled glass sand (R-G) for the same lime contents (R-G5, R-G10, and R-G15), with So/Sa ratios of 0.94, 0.97, and 0.96. The third group was produced using CDW as a substitute for both natural sand and coarse aggregates PG and LS (CDW5, CDW10, and CDW15), and the soil/CDW ratios (So/CDW) were 1.13, 1.01, and 0.90, respectively. Finally, the last group of mix designs was prepared to combine the two secondary materials in the same matrix, where the fraction of CDW was equal to the combined fraction of R-G plus PG and LS (R-GCDW5, R-GCDW10, and R-GCDW15). The combined fraction of soil/aggregates (So/Ag) was 1.23, 1.06, and 0.94. One study reported that the clay/sand ratio in their CEBs was correlated with their mechanical properties and water absorption, obtaining compressive strengths of 5.59 and 6.08 MPa when the clay/sand (So/Sa) ratios were 0.98 and 0.93, respectively [56], both close to most of the proportions calculated for the 12 mixtures in this research.

The statistical analysis and verification of correlations was determined by implementing the Statistical Software Package for Social Sciences (SPSS) v. 29.0.0.0 (241); it was considered that all the variables in this study were quantitative and continuous or ratio. Initially, to verify the correlation of the variables, the Shapiro–Wilk normality (*n* < 50) test was used with a significant value of α = 0.05 (5%) [57]; the correlations between variables were determined via the Pearson correlation coefficient (paired independent variables).

### 2.4. Experimental Campaign

#### Mixing

The mixing machine was selected so that it could produce a complete batch of each study variable. However, only by operating at under 25% of its theoretical capacity could it mix the heterogenous components with adequate uniformity. This was supported by a study mixing crushed grains in a similar mixer, concluding that the best indicators were obtained with a filling of 0.28 to 0.32 of the theoretical maximum volume [58]. In other research, it was also established that saturation of the theoretical maximum volume of the mixer causes a decrease in homogeneity [59].

This research used a Yardmax brand electric concrete mixer, model YM0115 (Roselle, IL, USA). All the aggregates were weighed on a digital scale with a Rubbermaid^®^ brand non-slip steel platform, measuring 30.5 × 31.8 cm, with a capacity of 68 kg and an accuracy of 0.09 kg.

**Figure 5:**
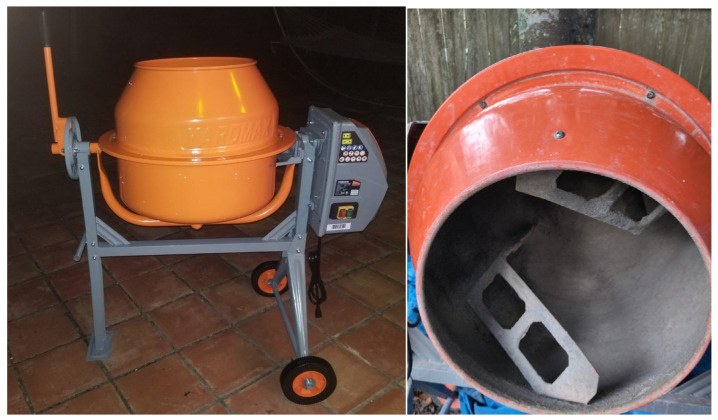
Mixing machine, exterior and interior view with the blades.

Since flocculation and aggregation are related to the adsorption of water on the surface of the clay particles [60], the following mixing order was established: first introduce the stone aggregates with the lime (depending on the mixture, they would be MRS/PG/LS/LIME, R-G/PG/LS/LIME, CDW/LIME, or R-G/PG/LS/CDW/LIME) and mix for two minutes. Add the SCS/SWD soils and the water, mixing for an additional four minutes. The total mixing time of each batch was six minutes, a duration similar to that of previous research [37,61,62]. Figure 5 shows the mixer (exterior and interior view).

### 2.5. Compressed Earth Block Making Machine

Each of the four mixed sub-batches was transferred to the hopper of the machine used to manufacture the CSEBs. The machine used was the “Ital Mexicana” brand, model Adopress 3000 (serial number 205199) a concept of the Cinva Ram [63].

Batch sizes ranged from 10 to 15 specimens each. T_0_ is defined as the condition of the mixture in the mold before the compressive force is applied, T_1_ when the CSEB comes out of the mold, and T_2_ at the end of the curing period.

**Figure 6:**
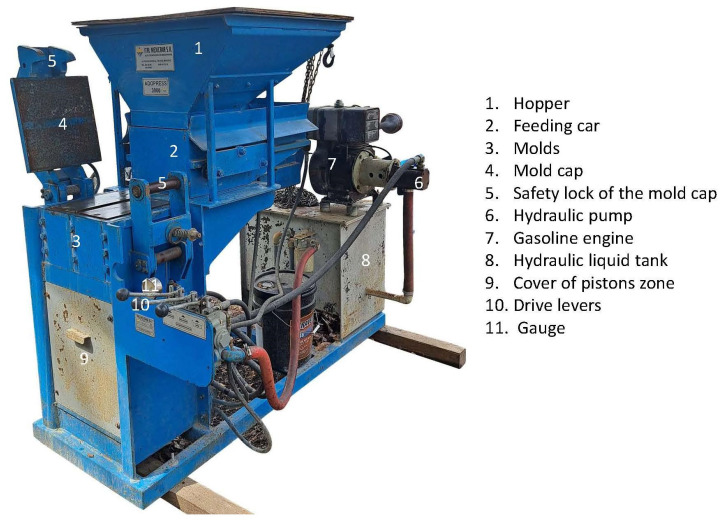
Adopress 3000—CEB Making Machine and its parts.

Figure 6 shows the machine parts.

#### 2.5.1. Curing

**Figure 7:**
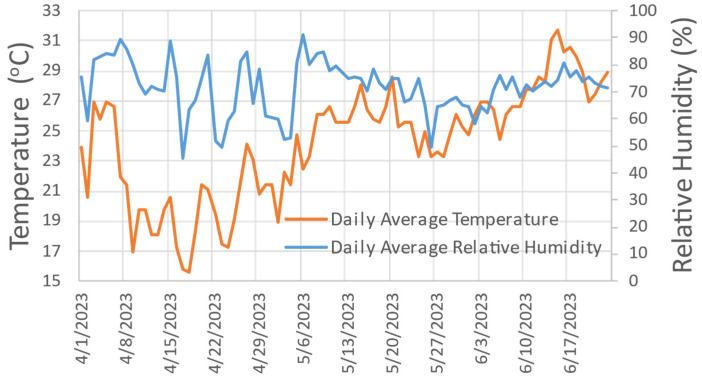
Temperature and relative humidity during the curing phase of the specimens.

**Figure 8:**
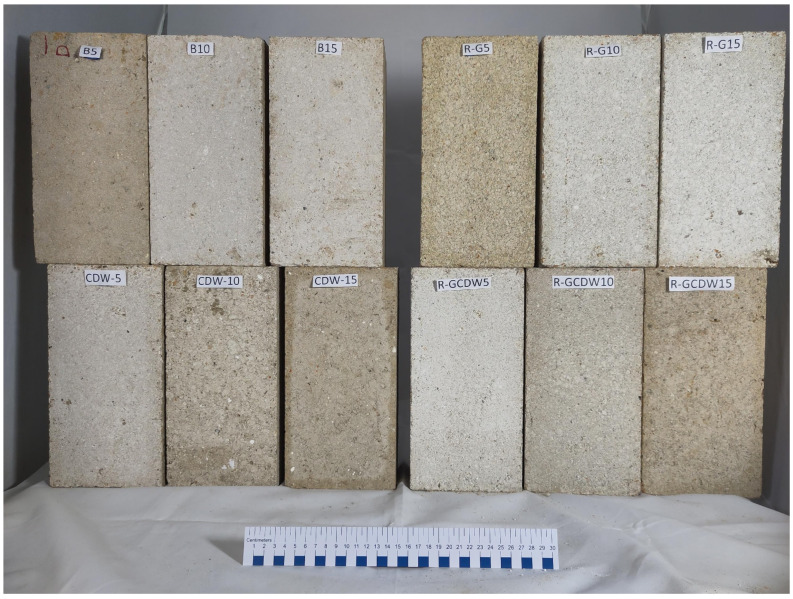
CSEB, one specimen per batch of the four matrices at T_2_, for size reference, the ruler has a total length of 30 cm.

The CSEBs were cured under environmental conditions in the city of Mandeville, Louisiana, USA, protected from rain. The target cure duration was 28 days [54,61,64,65]. The CSEB manufacturing run took place from 1 April to 23 June 2023, and each batch was made during a continuous 4- to 6-hour production shift, including all manufacturing activities. During the curing period, the minimum and maximum temperatures were 6 and 36 °C respectively, with an overall average of 32 °C [66,67,68]. Relative humidity (RH) ranged from 21 to 100%, with an overall average of 91% for the same curing period [66,67,68]. In previous studies, variants of the curing process have also been prescribed, showing how important it is to maintain a high RH during the first few days after molding (slow moisture loss); for example, CSEB curing periods were specified for only 14 days, but wrapped in plastic (to keep the RH high), followed by 14 more days of curing (drying phase) [64]. In another case, the RH of the air > 70% was also ensured by placing the CSEBs on a tray and covering them with a plastic sheet [61]. Figure 7 shows the temperature and RH during the curing period, while Figure 8 shows the final specimens at T_2_.

#### 2.5.2. Weight and Dimensions

Each specimen was weighed immediately after being demolded on a Suofei model SF-802 digital scale (Jiangyin, Jiangsu, China) with an accuracy of 1 g, a maximum supported weight of 25 kg, and a platform size of 21.5 × 17.5 cm. Each specimen’s width, length, and height were determined using a flexible metal tape measure with an approximation of one millimeter. This was repeated at T_2_.

#### 2.5.3. Compression Resistance Test

**Figure 9:**
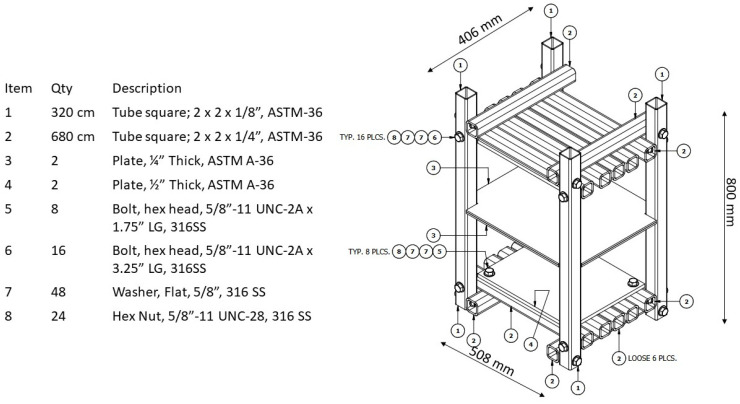
Construction diagram of the structural frame with its list of materials.

**Figure 10:**
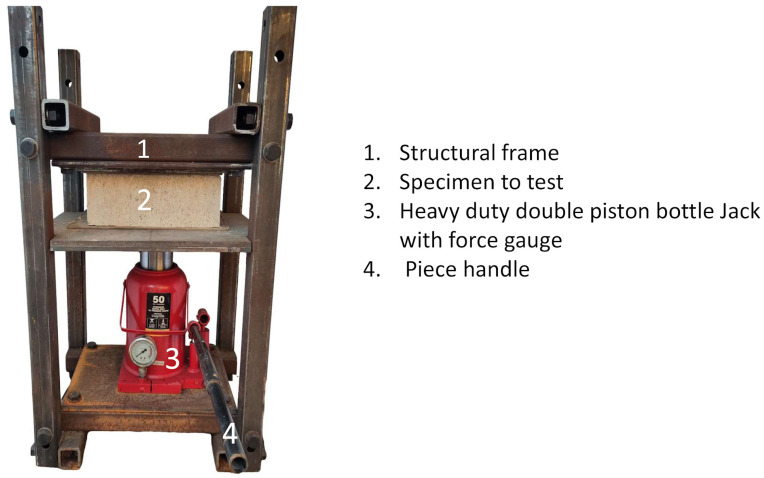
Field test apparatus to determine simple compressive strength in CSEB.

The simple compressive strength was measured with field equipment composed of a steel frame and a BIG RED Model ATH95000iBR hydraulic piston (Hebei, China), with a maximum capacity of 50,802.35 kg and equipped with a load meter. Previous studies have shown that this practice yields reliable results [69,70]. The compressive load was applied to the top face of the specimen (upper manufactured face) as specified in the NMX-C-036-ONNCCE-2013 standard [71]. The field equipment manufactured for this project was calibrated with a 400,000 lb. Tinius–Olsen Universal Test Set, belonging to the University of New Orleans. Figure 9 shows the design structure of the metal frame and a list of materials used for its manufacture, while Figure 10 presents an image of the system as a whole.

Two specimens were tested per batch of matrix (66% of what is specified in the regulations). For batches of 10, 12, and 14 CSEB specimens, the sample size equivalent to 20, 17, and 14% was consistent with the sample sizes reported in validating processes when destructive testing is required [72]. One of the tables reported in the previous paper was an adaptation of the sample plan introduced in ASTM-F302, which was also mentioned in another paper [73].

#### 2.5.4. Absorption Coefficient

**Figure 11:**
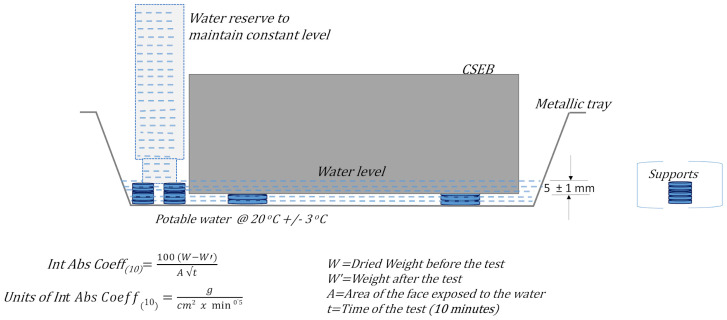
Schematic representation of the Int Abs Coeff_(10)_ test arrangement. Original drawing inspired by the three cited norms [74,75,76].

Following the same sample size criterion applied above, two CSEBs per batch of each study matrix were tested to determine the Initial Absorption Coefficient (Int Abs Coeff_(10)_). The test procedure applied was the combination of the provisions of the regulations NMX-C-037-ONNCCE-2013 [74], UNE 41410:2008 [75], and XP P 13-901-2001 [76]. The first two standards do not establish an acceptable upper limit, only indicating that it is prescriptive to report the value obtained from this test. However, in the case of the third regulation, it is specified that this property will not be taken into consideration for blocks intended for a dry environment. For blocks to be used on exterior walls, two limits are established: Int Abs Coeff_(10)_ ≤ 20 g/(cm^2^ × min^0.5^) for blocks classified as low capillarity and Int Abs Coeff_(10)_ ≤ 40 g/(cm^2^ × min^0.5^) for blocks classified as medium capillary. Figure 11 presents the configuration diagram of this applied test, as also indicated in the three standards cited.

## 3. Results

### 3.1. CSEB Linear Dimensions

The NMX-C-508-ONNCE-2015 standard [53] specifies that the variation of the effective dimensions of the block in relation to the nominal dimensions declared by the manufacturer must not exceed +/− 3 mm. Therefore, in each of the study matrices, the average dimensions were calculated and the variations in tolerances expressed as deviations from that average. Table 2 shows the variations or tolerances of these dimensions compared to the aforementioned standard.

The maximum values for the non-compliant units were ±5.75 mm.

**Figure 12:**
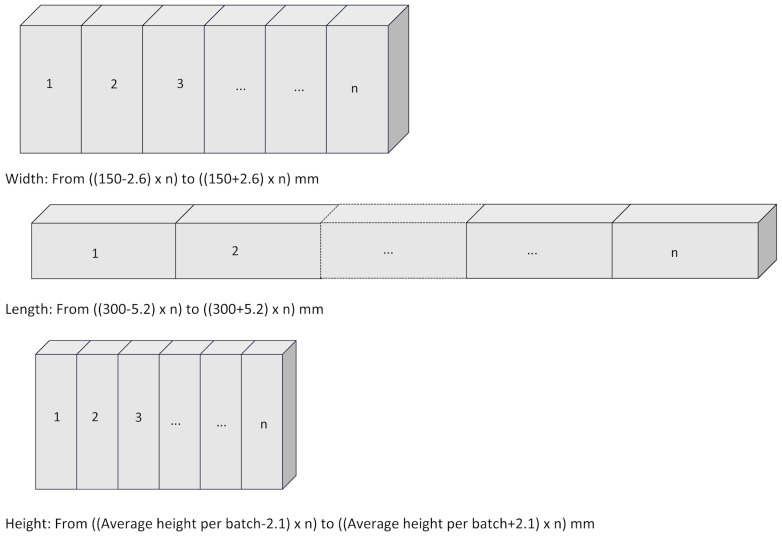
Linear tolerances, original drawing inspired by SI: 1077:1997 [80].

Other studies report the geometry of the blocks (width × length × height), but without mentioning if there was variation [77,78]. Another study analyzed the worldwide regulations regarding construction with earth, grouping them into 15 sets. It reports that the Brazilian, French, and Tunisian standards have scope for application on the CSEB geometry or dimensions without noting consequences in cases of deviations [79]. It can be considered that the impact of the variations in the CSEB dimensions would be on the walls to be built, since there may be a compensatory effect if some units were above or below the tolerances. This idea can be supported according to the tolerance established by the SI: 1077:1997 standard [80], which has been cited in another study [81] that indicates the tolerance for a linear dimension obtained from forming 20 fired bricks, where this line is repeated for each dimension. Although this test was not physically performed with the CSEBs in the present study, if this behavior is analyzed arithmetically by adjusting for each batch based on the number of units, all batches would comply with this behavior. Figure 12 illustrates this concept.

**Figure 13:**
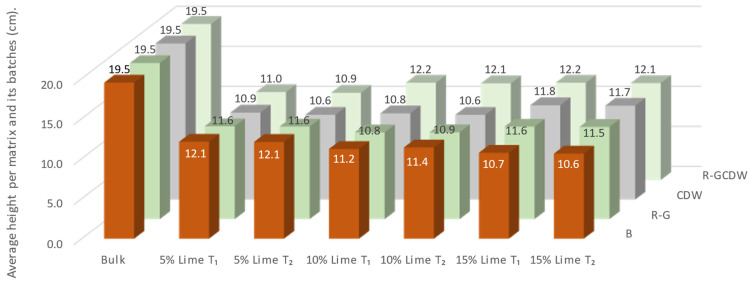
Height of the blocks determined by the bulk weight and the heights at T_1_ and T_2_.

The dimension that had the greatest variation between the different molds and batches was the height, which is determined by the compression of the specimen during the machine molding phase. The average height per T_2_ matrix ranged from 10.58 to 12.12 cm, obtaining an overall average of 11.34 cm for all the study matrices. The average outlet height before molding (T_0_) as a function of the apparent weight of each mixture was the same, due to volumetric dosing. Figure 13 shows the height prior to compression by the machine, then T_1_ and T_2_. The change in height before and after matrix curing was not significant (the coefficient of variation of average heights was 0.05 for both T_1_ and T_2_).

### 3.2. CSEB Weight

The average weight of the specimens per mixture production at T_2_ ranged from 8.56 to 10.10 kg, reaching an overall average of all the study mixtures equal to 9.21 kg. The R-G10 batch had the least change, losing 6% of weight during the curing process, while the CDW10 had the greatest change, losing 11.4%. 

On the other hand, when comparing the batches of each matrix of T_1_, matrix B had a decrease of 0.9 kg when increasing from 5 to 10% of lime and of 0.27 kg when going from 10 to 15%. The R-G matrix showed a decrease of 0.54 kg by increasing the lime from 5 to 10%, compared with an increase of 0.43 kg for 10 to 15% of lime. The CDW matrix behaved similarly, with a decrease of 0.25 kg when going from 5 to 10% of lime and an increase of 0.63 kg when increasing from 10 to 15% of lime. Finally, the R-GCDW matrix differs from the previous three because first there was an increase of 0.53 kg when going from 5 to 10% of lime, followed by a decrease of 0.15 kg when using 10 to 15% of lime. 

**Figure 14:**
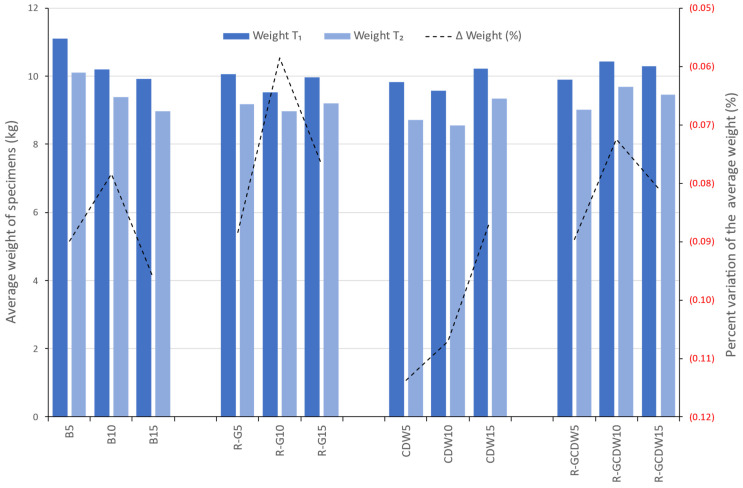
Average weights at T_1_ and T_2_ and the percentage variation.

At T_2_, all the matrices behaved the same as at T_1_, except that the values varied, as can be noted in Figure 14. This shows the average weight of the specimens by type of study matrix and its three batches at T_1_ and T_2_; likewise, the graph shows the percentage variation of the change in weight of the specimens on the secondary axis.

It is observed that the curing process has a drying effect since all average weights decreased from T_1_ to T_2_.

### 3.3. CSEB Volume

The volume for T_2_ ranged from 4752 cm^3^ to 5447 cm^3^, reaching a global average of 5094 cm^3^ for all matrices. For this determination, the measured dimensions were used (averaged for each batch). The volumetric variation from one matrix to another presents a greater difference compared to the weights; however, the variation expressed as a percentage is smaller, ranging from −3% to 1%.

When comparing T_1_, for each matrix and its batches, matrix B showed a decrease of 448 cm^3^ by increasing lime from 5 to 10% and of 254 cm^3^ by going from 10 to 15%. For the R-G matrix, there is a decrease of 344 cm^3^ when increasing lime from 5 to 10% but an increase of 321 cm^3^ when going from 10 to 15% lime. The CDW matrix behaved similarly, with a decrease of 41 cm^3^ from 5 to 10% lime and an increase of 451 cm^3^ from 10 to 15% lime. Finally, the R-GCDW matrix differs from the previous three as in both lime increases the volume also shows an increase; first of 515 cm^3^ when going from 5 to 10% and then of 11 cm^3^ when going from 10 to 15% of lime. 

**Figure 15:**
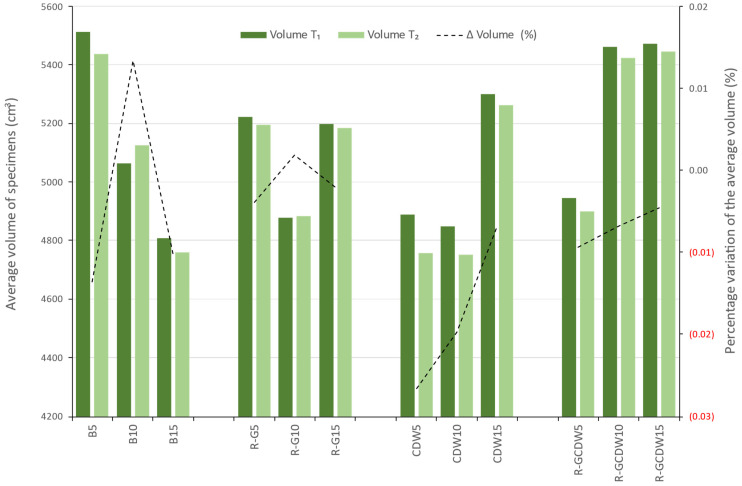
Average volume at T_1_ and T_2_ and the percentage variation.

At T_2_, all the matrices behaved the same as at T_1_, except that the values varied, as can be observed in Figure 15, which shows the volume averages for T_1_ and T_2_, with the graph showing the percentage change in specimen volumes on the secondary axis.

### 3.4. CSEB Density

Density was determined as the mass of each specimen divided by its volume. The range of average values obtained for all matrices at T_2_ was from 1736 to 1887 kg/m^3^, with an overall average of 1810 kg/m^3^. The base matrices B5, B10, and B15, as a matrix group, were denser after demolding and curing compared to the rest of the matrices.

When comparing T_1_, for each matrix and its batches, matrix B had an increase of 1.9 kg/m^3^ by increasing from 5 to 10% of lime and of 50.0 kg/m^3^ by going from 10 to 15%. On the contrary, for the R-G matrix, there is an increase of 24.5 kg/m^3^ when the lime is changed from 5 to 10% but a decrease of 37.8 kg/m^3^ when the lime is increased from 10 to 15%. The CDW matrix differs from the other two as it shows a decrease of 35.3 kg/m^3^ when going from 5 to 10% of lime and of 48.5 kg/m^3^ when increasing from 10 to 15% of lime. Finally, the R-GCDW matrix behaves similarly to the previous one, with a decrease of 91.0 kg/m^3^ when going from 5 to 10% lime and of 31.2 kg/m^3^ when going from 10 to 15% lime.

**Figure 16:**
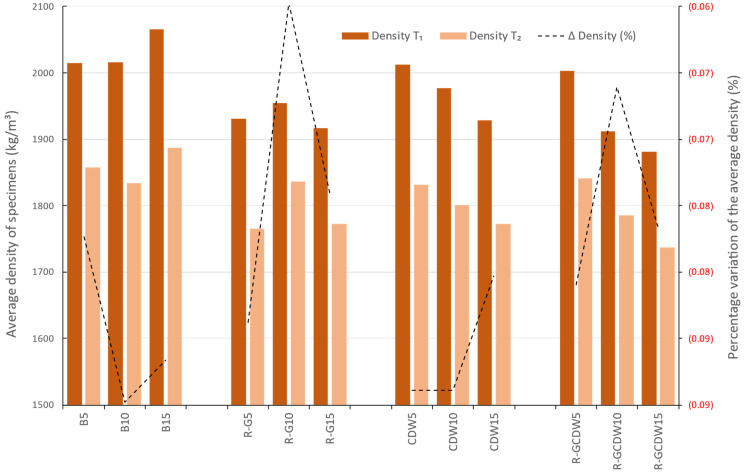
Average density at T_1_ and T_2_ and the percentage variation.

At T_2_, the decrease of 1.3% in matrix B would be considered negligible; it could also be said that the behaviors were the same, only with changes in the values. This can be noted in Figure 16, which shows the average density values of all the matrices studied and the percentage change.

### 3.5. CSEB Compression Resistance Test

The results of the compressive strength tests have been compared with the following three standards and their limits:

1. The USA-2015 International Building Code (IBC), applicable in several cities in southern Louisiana: 2.06 MPa [82].
2. Mexican Standard NMX-C-508-ONNCCE-2015 for Compressed Earth Blocks (BTC) 3: 2.94 MPa [53].
3. Spanish Standard UNE-41410-2008 for BTC 3: 3.00 MPa [75].

**Figure 17:**
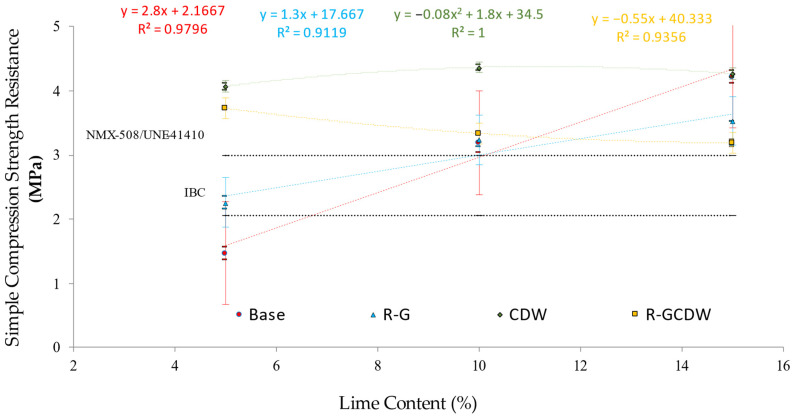
Results of simple compressive strength by matrix at different lime concentrations.

With regard to the minimum limits of the compressive strength standards of the base matrix, lot B5 was below the three reference standards, while B10 (with 7% more than the limit of the most restrictive standards) and B15 (42% above the limit of the most restrictive standards) did comply with the minimum limits of all standards. For the R-G matrix, lot R-G5 only exceeds the minimum IBC limit, while R-G10 and R-G15 meet the minimum limits of the three regulations, 9% and 19% more than the most restrictive limit, respectively. As far as the CDW matrix is concerned, the three batches exceed the three regulatory limits, being 37, 47, and 44% more than the most restrictive limits for CDW5, CDW10, and CDW15, respectively. Finally, in the case of the R-GCDW matrix, the three study batches also exceeded all regulations, with R-GCDW5, R-GCDW10, and R-GCDW15 being respectively 25, 12, and 7% more than the most restrictive limit. Figure 17 shows the results of the compressive strength tests for each matrix and its study batches (indicating in each batch the average value, the range of variation, and the typical error), as well as the three minimum limits accepted by the regulations and the regression equations and correlation coefficients R^2^.

Regarding the general behavior of the different matrices, the base matrix, the G-R matrix, and the R-GCDW all establish linear equations with significant high R^2^ correlation coefficients. This makes it possible to predict linear behaviors between the different variables of replacement contents of the materials in each study matrix, simply established predictive behaviors. In the case of the CDW matrix, a square-type adjustment is necessary to achieve an adjusted R^2^. This shows that in this matrix there is a change in the behavior between the variables of the studied batches, with the existence of a “hidden” variable not identified in the experimental campaign, which means the predictive behavior is not easily established. Although it is true, the scalar constant term of the variable in the second order is reduced (−0.008x^2^), which indicates that the unidentified effect has an impact on the behavior of the low variables, also denoted in the curve with a reduced slope. 

The statistical result for the Shapiro–Wilk normality test established a value of asymptotic or bilateral significance *p* ≥ 0.05 for all the correlations; therefore, normal distributions may be assumed (parametric tests). Therefore, the existing correlation between the study variables was determined using the Pearson correlation. Regarding these correlation results for the variables base, R-G, CDW, and R-GCDW in relation to the lime content (%), the following coefficients of correlation were obtained: 0.986 (p bilateral < 0.001), 0.948 (p bilateral < 0.001), 0.621 (p bilateral = 0.74), and −0.962 (p bilateral < 0.001). In the case of correlation CDW in relation to the lime content (%), its *p* value of significance is superior to α = 0.05; the latter could be interpreted as indicating that this correlation does not have a confidence interval (CI) = 95%, but nevertheless the existence of correlation should be considered.

The correlations cited above could be considered with a magnitude or force of correlation classified as strong or very strong (depending on the reference criteria [83]), with the exception of one CDW with regard to the lime content (%), which is moderate or strong. Regarding the type of correlation established, all are direct, with the exception of the correlation between R-GCDW in relation to the lime content (%), which is inverse.

With respect to similar previous works, there are compressive strength results reported by other studies that used lime as a stabilizer, with simple compressive strengths of 2.45 MPa [77], 2.65, and 4.71 MPa [84] mentioned. In a study that tested commercial CSEB from three factories (with a total of 25 specimens tested), an average strength of 1.57 MPa was obtained, with a range of variation between 0.26 and 3.86 MPa [70].

### 3.6. Initial Absorption Coefficient (Capillarity) for the CSEBs under Study

In the batches of the base matrix, the average value of the Int Abs Coeff_(10)_ (always in units of g/(cm^2^ × min^0.5^)) decreased directly with the increase in lime concentration: B5 = 57.7, B10 = 32.75, and B15 = 14.7. In the case of R-G, the Int Abs Coeff_(10)_ is also inverse to the increase in lime concentration: R-G5 = 36.6, R-G10 = 30.5, and R-G15 = 21.4. Regarding the CWD matrix, the Int Abs Coeff_(10)_ in the three batches studied continued to show similar values: CDW5 = 13.6, CDW10 = 12.7, and CDW15 = 13.1 (with 10% lime being the lowest). Similarly, the same trend of results was established for the R-GCDW matrix: R-GCDW5 = 19.45, R-GCDW10 = 20.9, R-GCDW15 = 18.45, although in this matrix the concentration of 10% lime reached the maximum value. In a previous study, matrices similar to those studied here (but with fiber additions of 1 to 20%) established average values of the Int Abs Coeff_(10)_ equal to 26 (in a range of variation from 29 to 59) [85].

**Figure 18:**
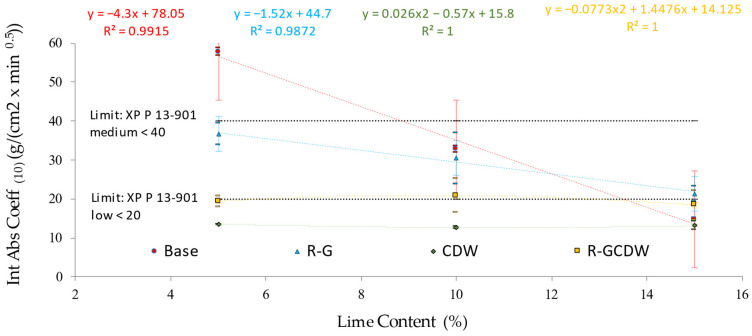
Int Abs Coeff_(10)_ results per matrix at different lime concentrations.

In the present study, according to the XP P 13-901-2001 standard [76], only B15 would comply with any of the limits established therein, meaning that 58% of the matrices of this research should be considered as CSEB of low capillarity. This is also analogous to what was reported in previous research. In this work, the lime content was studied in concentrations of between 8 and 12% (with fiber contents between 0 and 0.2%) [25]. Figure 18 shows the average value of the Int Abs Coeff_(10)_ for each matrix in relation to the percentage of lime included in each batch.

Regarding the general behavior of the different matrices, both matrix B and R-G indicate inverse linear equations with significantly high R^2^ correlation coefficients with respect to the lime content used in each matrix. As in the compressive strength property, the CDW matrix for the Int Abs Coeff_(10)_ property shows behavior that requires quadratic fitting—consistency between the properties of the same matrix results. However, the R-GCDW matrix for this property, Int Abs Coeff_(10)_, has required a quadratic fit resembling the CDW matrix (but with a negative sign).

The statistical result of the Shapiro–Wilk normality test established a value of asymptotic or bilateral significance *p* ≥ 0.05 for all the correlations; therefore, normal distributions may be assumed (parametric tests).

Regarding the results of Pearson’s correlation for the studied variables base, R-G, CDW, and R-GCDW in relation to the lime content (%), the following coefficients of correlation were obtained: 0.995 (p bilateral < 0.001), −0.869 (p bilateral = 0.002), −0.318 (p bilateral = 0.405), and −0.137 (p bilateral = 0.725). In the case of correlation CDW and R-GCDW in relation to the lime content (%), its value of significance p is superior to α = 0.05, which can be interpreted as saying that this correlation does not have a confidence interval (CI) = 95%; nevertheless, the existence of correlation should also be considered.

The correlations cited above could be considered with a magnitude or correlation force classified as strong or very strong in the cases of base and R-G, weak for CDW, and weak or negligible for R-CDW (always in relation to lime content (%)). Regarding the type of correlation that is established, all are inverse.

## 4. Discussion

It was established as a priority criterion for this research that in the applied experimentation the use of the semi-automatic characteristics of the equipment for manufacturing the CSEB (Adopress 3000) was allowed, and that the use of the materials and the curing phase of the CSEB were in uncontrolled environmental conditions (only safeguarded from rain). The three previous requirements limit this study when compared to others that, for example, dry soils before mixing [17,49], check that the weight of the material resulting from the mixture is constant before proceeding to compression molding [17,49], and maintain the environmental temperature and RH conditions during the curing phase [16], as well as the conditions under which CSEBs are produced in small or large factories for use in construction projects [38].

Therefore, the chosen approach faces the challenge of understanding the various factors that contribute to the characterization of each matrix, and while assuming this risk also seeks the potential for a greater gain: to be able to advance in the understanding of variability that will facilitate the transfer of theoretical academic technology to society in general. This empowerment of knowledge by society will lead to the possibility of constructing housing with CSEB in regions where earthen construction practices have distanced themselves from those that were once in force [86].

To produce the CSEBs using the manufacturing equipment, each mixture was loaded into the hopper, from where the feeder carriage supplied a constant volume to the mold for each unit. Therefore, it is assumed that each unit of CSEB had the same bulk density (established by the constant volume of the mold, 8775 cm^3^). Since the initial volume was the same for all units, the variation in the weight of the units is determined by the variation in the composition of the specimens and the rheology inherent in each batch of the matrices.

With regard to the compressive force (up to 14.71 MPa, maintained for three seconds), the accuracy of this requirement depends on the operator’s vision and attention to the dial, as well as an audible indication produced by a change in the noise of the pump. Therefore, in order to avoid variations or production biases, all samples were carried out by the same operator.

Once the compression step of the mixture has been carried out, the change in volume depends mainly on the variation in height, which is the direction in which the aforementioned step is performed. Since the other two dimensions (width and length) have a negligible variation, this can be supported if the percentage of the change is observed, i.e., the percentage of the dimension with respect to the dimension of the mold. The Matrix B batches with lime concentrations of 5, 10, and 15% had a height variation of 38, 43, and 45%, while the length variation was 1.07, 0.36, and 0.22%, as well as a variation in width of 0.07, 0.04, and 0.22%, respectively. These percentages for the R-G matrix were: height variation of 40, 44, and 41%, while length variation was 0.17, 0.00, and 0.06% and width was 0.22, 0.03, and 0.03%, respectively. In the CWD matrix they were: height variation of 44, 45, and 40%, while the variation in length was 0.00, 0.00, and 0.06%, and in width 0.00, 0.00, and 0.11%, respectively. Finally, the R-GCWD matrix showed a height variation of 44, 38, and 38%, while the variation in length was 0.00, 0.07, and 0.06%, as well as 0.00, 0.13, and 0.03% in width, respectively.

In previous works it has been reported that, with regard to the gravimetric feeders of CSEB factory equipment, the variations in the specimens produced are due to the variations in the densities of the materials caused by the change in the level of the material in the hopper during feeding [87]. This is applicable to a volumetric feeder such as the one used in this research since, in the procedure followed, the volume of the materials was controlled and, as a result, variations were established in the weights of the different specimens as well as in the average weights between each study matrix. The dosing of granular materials is further affected by other factors, such as the shape and size distribution of their own particles [88]. Each batch and matrix studied will have a different particle size distribution, which depends not only on the composition of the particles but also on the association between the components during mixing. This explains the variations in unit weight and average in the molds for a constant bulk volume. The highest average weight, 11.1 kg, was found in lot B5. In matrix B, as the percentage of lime increased, the unit weight of the specimens decreased (the average unit weight of B10 = 10.2 and that of B15 = 9.9 kg). The above pattern is evident, as the bulk density of lime is the smallest of all the materials used; however, this pattern is not observed in the rest of the matrices studied.

Regarding the volumes of the compressed specimens, the matrices with the highest average volume were the least compressed. The highest average volume of the specimens was for batch B5 with 5512 cm^3^, while the smallest average volume was established for batch B15 with 4809 cm^3^. This implies that the remaining batches and matrices of this study established average values of volumes among them. In previous studies, it has been reported that the compressibility of materials is directly related to the forces that occur between particles of various types [88]. When it is indicated that the particle size of the aggregates in each matrix batch is also inherent in the associations between the particles, it is feasible to think that some of these associations may be a consequence of the so-called “balling” effect (the formation of almost spherical granules by rotating moistened particles) [89]. This was not within the initial objectives or scope of this research as a parameter that would explain the behavior of the different batches and mixtures, and therefore there is no formal determination of the scope of this effect. However, it appears that reporting this observation is important for showing the source of the particle sizes that affect dosage. In Figure 8, a different granularity can be observed in each of the photographed blocks (also known as surface heterogeneity) [55].

Previous studies have established that simple compressive strength in CSEBs is significantly related to dry density at the compaction stage of specimens [90]. In this research, this is consistent with what was observed in two batches of different matrices, B15 and CDW5, while in the rest of the matrices with lower densities they achieved a higher compressive strength. It has been shown that the simple compressive strength is significantly linked to the distribution of the particle sizes of the materials used (which changes if the particle size profiles are changed), even for the same amount of the binder used [89]. This may help to partly explain the variability of compressive strength and the lack of a “universal” standard as the lime binder increases in the different batches of the study matrices.

In the case of the Int Abs Coeff_(10)_, the increase in lime causes a decrease in the coefficient in three batches of the matrices studied: B15, R-G15, and R-GCDW15. For the CDW matrix, lot CDW10 obtained the lowest coefficient, but for the three batches of this matrix, including CDW10, the values of the Int Abs Coeff_(10)_ obtained a standard deviation of 0.45 with an average of 13 g/(cm^2^ × min^0.5^). Consequently, it is possible that the same condition of the distribution of particle sizes (after mixing) affects the result, and in this case aspects such as pore size and surface open porosity are important for the Int Abs Coeff_(10)_. Therefore, it would depend on this distribution of particle sizes and the resulting pore size and surface accessibility, which affect water retention [81].

Table 3 presents the results of the simple compressive strength tests, the Int Abs Coeff(10), the variation of heights after compression, and the densities, all of which are average values per matrix batch studied. The same table also indicates the order achieved by each variable among all the study batches, as well as whether the variable manages to comply with the different applicable regulations.

**Figure 19:**
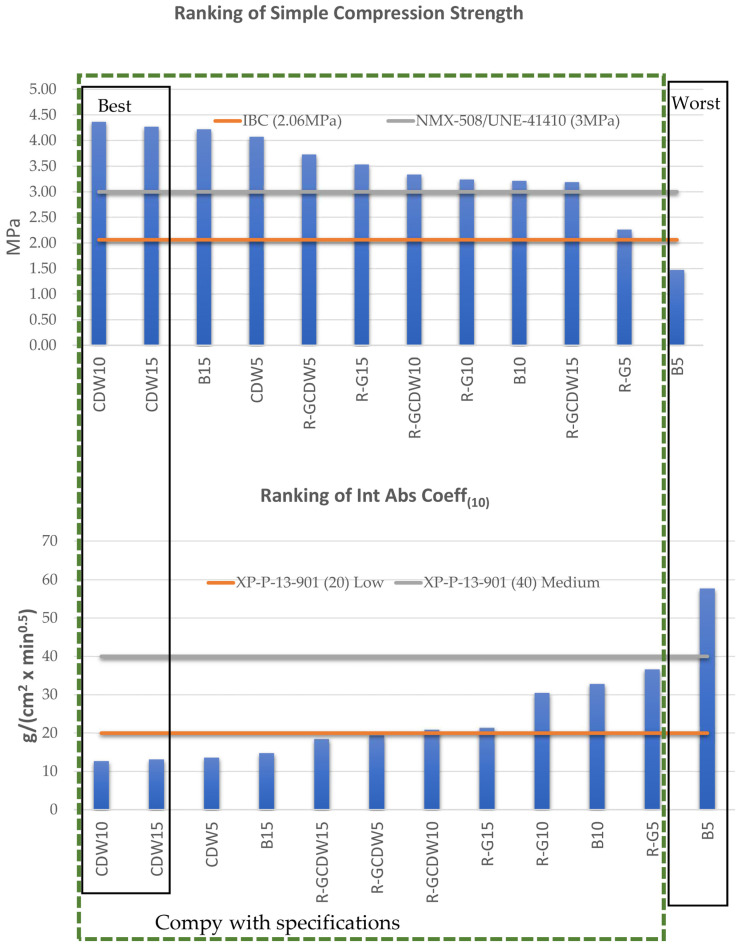
Ranking results of simple compression strength and Int Abs Coeff_(10)_.

For the two main tests of the study, simple compressive strength and Int Abs Coeff_(10)_, two batches of the CDW matrix are the best performing: CDW10 and CDW15, respectively. The rest of the study matrices, with the exception of batch B5, manage to satisfy the limits established by the reference regulations. It is necessary to show that there is a clear inverse correlation between the results of both tests: simple compressive strengths with high values produce low Int Abs Coeff_(10)_, and in both cases the density of the materials seems to be the link that connects them. Figure 19 shows the classification and order obtained for the study batches, a green rectangle with a dashed line indicating the batches that manage to meet the regulations, and a black rectangle showing the best/worst positioned batches with respect to the hierarchical order achieved in the tests.

The three batches of the CDW matrix were situated among the best in this research, which is consistent with previous studies that have shown that recycled aggregates from concrete provide significant resistance and improve the bond between particles [16], as well as being able to create resistant microstructures [17]. As regards the eight batches positioned between the optimal and deficient extremes, they do not follow a consistent classification in the two study tests and with reference to the type of the study batch, but they are shown to do so by comparing the performances of the two study tests.

The batches where MRS river sand was replaced with R-G with 10 and 5% lime outperformed base mixes for both parameters. No other studies were found that used this material for CSEB as a solid aggregate. However, in a study that dissolved crushed glass in sodium hydroxide, it was reported that the formation of a homogeneous gel increased the compressive strength of the specimens studied [91]. In another study in which ground waste glass was used for cement production, it was estimated that waste glass, ground to a microscale particle size, undergoes pozzolanic reactions [92].

## 5. Conclusions

Second-generation materials such as demolition waste from concrete and recycled glass sand can be used in dual mixtures, with the soil types studied in this research, to produce lime-stabilized CEBs subjected to uncontrolled RH and temperature conditions (wet and hot climate) with a mechanical compression of 14.72 MPa that will exceed the compressive strength requirements set by IBC [82], UNE-41410 [75], NMX-C-508-ONNCCE [53] for class 3 CSEB, and XP P 13-901 [76] for Int Abs Coeff_(10)_.

It is recommended to limit the lime content to 10% because, even in cases where the Int Abs Coeff_(10)_ and compressive strength are better with a lime content equal to 15% (maximum content specified by NMX-508 [53]), the improvement in these properties may not justify the extra 50% of stabilizer. 

All of the above would improve the sustainability of the current use of CSEB. It is possible to determine simple compressive strength with a simple field machine that would allow quality control of CSEB batches produced on-site.

It is also possible to make CSEB in situ in hot and humid climates without the need for complex structures or buildings with air conditioning.

A future phase of this research will be to determine the elemental compositions of the aggregates used, as well as the resulting matrix itself, in order to understand their impact on the behavior of the resulting CSEBs so as to better predict the possible interactions of these complex matrices.

## Figures and Tables

**Table 1:** Composition of each matrix and its three batches expressed in percent in weight.

Material (%)	Base Mix River Sand			Sand Replacement			Sand and Stone Replacement			Secondary Materials (R-G and CDW)		
	B5	B10	B15	R-G5	R-G10	R-G15	CDW5	CDW10	CDW15	R-GCDW5	R-GCDW10	R-GCDW15
Lime	5	10	15	5	10	15	5	10	15	5	10	15
Water	10	9	11	9	9	11	14	15	14	12	12	13
SCS	16	16	15	17	17	15	21	19	17	23	20	18
SWD	16	16	15	17	17	15	21	19	17	23	20	18
MRS	38	36	31	-	-	-	-	-	-	-	-	-
R-G	-	-	-	37	35	31	-	-	-	13	14	13
CDW	-	-	-	-	-	-	38	37	38	19	19	19
PG	7	7	7	7	6	7	-	-	-	3	3	3
LS	7	7	7	7	6	7	-	-	-	3	3	3
TOTAL	100	100	100	100	100	100	100	100	100	100	100	100

**Table 2:** Variation of dimensions of specimens in accordance with norm.

Matrices and Batches	Width	Length	Height
BS	AUP	AUP	30% ET
B10	AUP	AUP	AUP
B15	8% ET	AUP	33% ET
R-G5	AUP	AUP	25% ET
R-G10	AUP	AUP	55% ET
R-G15	AUP	AUP	17% ET
CDW5	AUP	AUP	AUP
CDW10	8% ET	AUP	AUP
CDW15	AUP	AUP	8% ET
R-GCDW5	AUP	AUP	AUP
R-GCDW10	AUP	AUP	AUP
R-GCDW15	AUP	AUP	9% ET

AUP: All units passed. ET: Percentage that exceeded tolerance.

**Table 3:** Average results for each matrix and its batches of main characterization parameters, with a ranking grade.

Batch/Matrix	Int Abs Coeff_(10)_, g/(cm^2^ × min^0.5^). Rank from Lowest to Highest	Simple Compression Strength Resistance (MPa)	Height Reduction as an Effect of Compression (%)	Dry Density T_1_ (kg/m^3^)	Dry Density T_2_ (kg/m^3^)
		Rank from Higher to Lower			
B5	$57.7_{{\#}_{12}}^{*}$	$1.472_{{\#}_{11}}^{*}$	$37.90_{{\#}_{10}}$	$2014_{{\#}_{3}}$	$1858_{{\#}_{2}}$
B10	$32.8_{{\#}_{10};\;MC}$	$3.188_{{\#}_{9}}^{**}$	$42.53_{{\#}_{7}}$	$2016_{{\#}_{2}}$	$1833_{{\#}_{5}}$
B15	$14.7_{{\#}_{4};\;LC}$	$4.218_{{\#}_{3}}^{**}$	$44.96_{{\#}_{1}}$	$2066_{{\#}_{1}}^{*}$	$1887_{{\#}_{1}}^{*}$
R-G5	$36.6_{{\#}_{11};\;MC}$	$2.256_{{\#}_{10}}^{***}$	$40.47_{{\#}_{8}}$	$1930_{{\#}_{8}}$	$1766_{{\#}_{10}}$
R-G10	$30.5_{{\#}_{9};\;MC}$	$3.237_{{\#}_{8}}^{**}$	$44.43_{{\#}_{4}}$	$1955_{{\#}_{7}}$	$1836_{{\#}_{4}}$
R-G15	$21.4_{{\#}_{8};\;MC}$	$3.532_{{\#}_{6}}^{**}$	$40.77_{{\#}_{2}}$	$1917_{{\#}_{10}}$	$1773_{{\#}_{9}}$
CDW5	$13.6_{{\#}_{3};\;LC}$	$4.071_{{\#}_{4}}^{**}$	$44.27_{{\#}_{5}}$	$2012_{{\#}_{4}}$	$1832_{{\#}_{6}}$
CDW10	$12.7_{{\#}_{1};LC}$	$4.365_{{\#}_{1}}^{**}$	$44.74_{{\#}_{3}}$	$1977_{{\#}_{6}}$	$1801_{{\#}_{7}}$
CDW15	$13.1_{{\#}_{2};\;LC}$	$4.267_{{\#}_{2}}^{**}$	$39.57_{{\#}_{9}}$	$1928_{{\#}_{9}}$	$1773_{{\#}_{9}}$
R-GCDW5	$19.4_{{\#}_{6};\;LC}$	$3.728_{{\#}_{5}}^{**}$	$43.63_{{\#}_{6}}$	$2003_{{\#}_{5}}$	$1841_{{\#}_{3}}$
R-GCDW10	$20.9_{{\#}_{7};\;MC}$	$3.335_{{\#}_{7}}^{**}$	$37.64_{{\#}_{11}}$	$1912_{{\#}_{11}}$	$1785_{{\#}_{8}}$
R-GCDW15	$18.4_{{\#}_{5};\;LC}$	$3.188_{{\#}_{9}}^{**}$	$37.62_{{\#}_{12}}$	$1881_{{\#}_{12}}$	$1736_{{\#}_{11}}$

Ranking of average values for each parameter is indicated with the subscript. Best starts with #1. * Results that did not satisfy any norm; ** Results compliant with the following norms: IBC [82], UNE [75], and NMX [53]; *** Result compliant with only IBC [81]; *MC:* compliant with medium capillarity [76]; *LC:* compliant with low capillarity [76].

## Data Availability

In the case of justified request, supporting information for this research can be requested from the corresponding author.
